# Incidence of uterine infections, major bacteria and antimicrobial resistance in postpartum dairy cows in southern Ethiopia

**DOI:** 10.1186/s12866-023-03160-w

**Published:** 2024-01-03

**Authors:** Berhanu Mekibib, Mesfin Belachew, Biruhtesfa Asrade, Girma Badada, Rahmeto Abebe

**Affiliations:** 1 Faculty of Veterinary Medicine, Hawassa University, P.O.Box 05, Hawassa, Ethiopia; 2 Dunna District Agriculture Office, Hadiya Zone, Central Ethiopia Region Ethiopia; 3 Collegeof Veterinary Medicine and Agriculture, Addis Ababa University, P.O.Box 34, Bishoftu, Ethiopia; 4 School of Veterinary Medicine, Ambo University, P.O.Box 19, Ambo, Ethiopia

**Keywords:** Antimicrobial resistance, Bacterial isolation, Dairy cows, Uterine infection

## Abstract

**Background:**

Uterine infections, primarily caused by bacterial pathogens, pose a significant problem for dairy farmers worldwide, leading to poor reproductive performance and economic losses. However, the bacteria responsible for uterine infections have not been adequately studied, nor has the antibiotic susceptibility of the causative bacteria been frequently tested in Ethiopia. This study aims to estimate the cumulative incidence of uterine infections in postpartum dairy cows, identify bacterial causes and determine antimicrobial susceptibility profile of the isolated bacteria.

**Methods:**

A prospective cohort study was conducted in which 236 cows from 74 dairy farms were monitored biweekly from calving to 90 days postpartum for metritis, endometritis and other disorders. Aseptic uterine swab samples were collected from 40 cows with uterine infections. The samples were cultured, and the isolated bacteria were tested for antimicrobial susceptibility using the disk diffusion method.

**Results:**

Out of 236 cows monitored during the postpartum phase, 45 (19.1%) were found to have contracted uterine infection. The cumulative incidence of metritis was 11.4% (*n* = 27), while the cumulative incidence of endometritis was 7.6% (*n *= 18). Of the 40 cultured swab samples, 29 (72.5%) had one or more bacteria isolated. The most commonly isolated bacteria were *Escherichia coli* (45%), coagulase-positive staphylococci (30%), and *Klebsiella* spp. (22.5%). Other bacterial spp, including *Arcanobacterium pyogenes* (12.5%), *Fusobacterium* spp. (12.5%), *Enterobacter aerogenes* (12.5%), coagulase-negative staphylococci (12.5%), *Streptococcus* spp. (7.5%), *Salmonella* spp, (5%) *Proteus* spp (5%) and *Pasteurella* spp (2.5%) were also isolated. All of the isolated bacteria demonstrated resistance to at least one of the antimicrobials tested. Multidrug resistance was observed in *E. coli*, *Klebsiella* spp., *A. pyogenes*, and *Fusobacterium* spp. Gentamicin was found to be the most effective antimicrobial against all bacteria tested, while tetracycline was the least effective of all.

**Conclusion:**

The study found that a significant proportion of cows in the population were affected by uterine infections and the isolated bacteria developed resistance to several antimicrobials. The study emphasizes the need for responsible use of antimicrobials to prevent the emergence of antimicrobial resistance. It also highlights the importance of raising awareness among dairy farmers to avoid the indiscriminate use of antibiotics and its consequences.

## Introduction

Ethiopia has a huge potential in the dairy industry, with an estimated 65 million cattle [1]. However, dairy production in Ethiopia is mostly extensive, focusing mainly on subsistence and characterized by low production and productivity [2]. Nevertheless, there is currently a rapid increase in dairy farms in urban and peri-urban areas in the country due to the growing demand for dairy products, driven by urbanization and population growth. Urban and peri-urban dairies are semi-intensive to intensive production systems that maintain exotic and cross-bred cows with comparatively better management practices [3]. Although the dairy industry has grown significantly in recent years, it faces numerous constraints, including diseases such as uterine infections, among others.

Uterine infections, such as endometritis or metritis, in dairy cows, can have significant economic consequences for dairy farmers. These infections result in reduced milk production, decreased reproductive efficiency, increased veterinary costs, and higher culling rates due to infertility [4]. In developed countries, uterine infection remains one of the most expensive diseases, posing major challenges to the dairy industry, costing approximately $650 million annually in the United States and €1.4 billion in Europe [5]. Considering these numbers, it is reasonable to assume that the impact of uterine infections on the emerging dairy industry in Ethiopia could be enormous due to the lack of prevention and control practices in place. Although there are numerous predisposing factors for uterine infection in dairy cows, the most important factors include parity, calving difficulty, retained placenta, twins, stillbirths, male offspring, ketosis, hypocalcemia, compromised immune status, and excessive growth of pathogenic microorganisms in the reproductive tract [6, 7].

Uterine infections can be caused by different types of bacteria, including *Escherichia coli*, Streptococcus species, *Trueperella pyogenes*, *Fusobacterium necrophorum*, and *Prevotella melaninogenica*, as reported by Bicalho et al. [8] and Williams et al. [9]. To manage and treat uterine infections, antimicrobial drugs have been widely used, especially in developing countries [10]. However, the indiscriminate use of antibiotics for treating and preventing uterine infections has led to the development of antimicrobial resistance (AMR) in bacteria causing these infections, which has become a growing concern globally [11]. Such antimicrobial resistant organisms could pose significant health risks to both animals and humans [12].

Several authors have reported the occurrence of uterine infections, particularly metritis and endometritis, in dairy farms in Ethiopia [13–15]. However, the bacteria responsible for uterine infections have not been adequately studied, nor has the antibiotic susceptibility of the causative bacteria been frequently tested. The AMR of bacteria in the uterine fluid of postpartum dairy cows is poorly described in Ethiopia. There is only a single published study in the country describing AMR of bacteria isolated from dairy cows with uterine infection [13]. It is known that reproductive disorders such as metritis and endometritis can significantly impact the reproductive performance and overall productivity of dairy cows. Therefore, it is crucial to understand the incidence of these diseases and the most commonly incriminated bacteria in different countries in order to develop targeted interventions and management strategies for effective prevention and control [7]. Therefore, this study was conducted to estimate the cumulative incidence of uterine infection in dairy cows, identify the commonly incriminated bacterial population, and investigate the sensitivity of the isolated bacteria to the commonly available antimicrobials.

## Materials and methods

### Study area

The study was conducted between December 2020 to December 2021on dairy farms located in Hawassa, Wolaita Sodo and Arsi Negelle towns. These areas were selected because of their relatively large population of dairy cattle and accessibility. Additionally, they are regarded as high potential areas for milk production in the southern Ethiopian milk shed. According to the Department of Livestock and Fisheries Development (2020) of each municipality, there are about 107 dairy farms in Hawassa, 35 in Arsi Negelle, and 33 in Wolaita Sodo. Herd sizes ranged from 2 to 131 cattle, with an average of 7 cattle per herd.

Hawassa, the capital of the Sidama Regional State, is situated 275 km south of Ethiopia’s capital, Addis Ababa. The city is located at an altitude of 1708 m above sea level and at 7^0^ 3' N latitude and 38^0^ 29' E longitude. Its annual temperature ranges from 20.1 to 25 °C, with rainfall varying from 800 to 1000 mm. Arsi Negelle town, which is found in the West Arsi zone of the Oromia regional state, is 225 km away from Addis Ababa. The town is approximately 2043 m above sea level at latitude 7^0^ 21' N and longitude 38^0^ 42' E. It experiences an average annual temperature of 10–25 °C, with rainfall ranging from 500 to 1000 mm. Wolaita Sodo, a town in the Southern Nations, Nationalities, and Peoples Regional State, is located 329 km south of Addis Ababa at an altitude of 700 to 2950 m above sea level. It is situated between 6º4´N to 7º1´N and 37º4´E to 38º2´E. The average annual rainfall in the town is between 450 and 1446 mm. The town’s average annual maximum and minimum temperatures are 34.12 and 11.4 °C, respectively [1].

### Study population

The study population consists of postpartum dairy cows in urban and peri-urban dairy farms found in the aforementioned study areas. Small to large scale dairy farms are springing up in these areas due to rapidly growing human population, as well as increased urbanization and demand for dairy products. Dairy production in these areas varies from the extensive traditional type to semi-intensive to intensive commercial milk production. The extensive production system is largely dependent on indigenous breeds, while the semi-intensive and intensive dairy farms tend to breed high-performing cross-bred or exotic cows. Our study focused on intensive and semi-intensive dairy farms. On intensive farms, the cattle were kept in tie stalls all the time and fed concentrates and roughages. On semi-intensive farms, the animals are housed in free stalls, the cattle grazed outside during the day and only received supplementary feed in the morning and evening shortly before milking. Most dairy farms had Holstein–Friesian local crosses, with some pure Jersey breeds and local zebu breeds in only three farms each. Artificial insemination (AI) has been the most commonly used method by the dairy farmers to breed cows. However, if conception failed after the AI or the AI technician was late, all farms would use the bull service as a backup.

### Study design, sample size and sampling technique

A prospective cohort study design was used to generate the data required for the study. The list of dairy farms in each town was identified in consultation with the veterinary staff of each town’s Livestock and Fisheries Resources Office. From the list of dairy farms, those with 5 or more cows were purposively selected, resulting in 74 dairy farms. In the selected farms, all cows that calved during the study period (236 cows in total) were monitored for uterine infections every two weeks from calving date until 90 days postpartum. Cows with visible uterine discharge, with or without a history of abnormal calving status (such as dystocia, abortion, retained fetal membrane, vaginal prolapse, or a combination thereof) were chosen for bacterial isolation and characterization. None of the cows had received intrauterine/parenteral antibiotics or reproductive hormones within two days prior to sampling. The study included all available breeds of dairy cattle at different parity levels.

### Field observation and physical examination

Each farm was visited biweekly to check for any postpartum events, such as retained fetal membrane, metritis, or endometritis. All clinical signs and relevant history regarding postpartum problems were also documented. Cows with uterine discharge underwent further examination of their vagina and cervix using a sterile vaginal speculum to determine the type and nature of the discharge. The discharge was categorized as clear mucus, predominantly clear mucus with traces of pus, mucopurulent (approximately 50% pus and 50% mucus), purulent (> 50% pus) but not foul-smelling, or purulent or red-brown and foul-smelling based on the classification by LeBlanc et al. [16]. Generally, cows with recent reproductive disorders and/or clinical symptoms underwent comprehensive examinations, and all findings, including rectal temperature, respiration, and pulse rate, were recorded.

### Case definition

A cow with red-brown watery foul-smelling uterine discharge and systemic signs of illness, including fever, dullness, loss of appetite and low milk production occurring within 21 days after calving, was diagnosed as having metritis. On the other hand, cows without any systemic illness but with muco-purulent or purulent uterine discharge occurring 3 weeks after parturition were diagnosed as cases of endometritis [4, 17].

### Sample collection and microbiological examinations

After cleaning the perineum with soap and water, the vulva was disinfected using tincture of iodine. A sterile vaginal speculum was employed to guide the swab. Trans-cervical guarded swab samples were collected from the uterine body of each cow that exhibited signs of uterine discharge between 5 to 30 days postpartum. The collected swab samples were placed into Stuart transport medium and transported in an ice box to the Hawassa University Veterinary Microbiology Laboratory for microbiological examination. Upon arrival, all samples were directly streaked on blood agar plates (Oxoid) containing 5% defibrinated sheep blood and MacConkey agar plate to obtain single colonies (primary isolates) side-by-side. If immediate inoculation was not feasible, the samples were kept at 4 °C until cultured within1 to 4 days for isolation. The inoculated/streaked plates were then incubated aerobically at 37 °C and checked for bacteriological growth after 24 – 72 h of incubation.

Bacterial colonies were identified based on colony characteristics, Gram stain, hemolytic properties, and growth on selective media. Following the initial identification, culture-specific biochemical tests and standard tests were conducted. Additionally, the cultures underwent primary identification tests such as the catalase test, as well as secondary biochemical tests including mannitol fermentation, pigment production, IMViC tests (indole test, methyl red test, Voges-Proskauer test, Citret utilization test), growth on eosin methylene blue agar (EMB), motility test on SIM medium, and coagulase test [18].

### Antibacterial susceptibility testing

The antibiotic susceptibility tests of the bacterial isolates were performed using Kibry-Bauer disk diffusion test on Muller Hinton agar (HIMEDIA, Mumbai) based on Clinical and Laboratory Standards Institute protocols [19]. Six antibiotic diffusion discs, namely gentamicin (G10μg/disc), amoxicillin (Am20μg/disc), chloramphenicol (C30μg/disc), tetracycline (T10 μg/disc), ampicillin (AMP25 μg/disc), and streptomycin (S10 μg/disc). These antimicrobial agents were chosen based on clinical considerations, taking into account their frequent use in the study area and availability on the market.

Pure culture colonies were transferred to a test tube, suspended in 5 ml of peptone, and incubated at 37 °C for 24 h. The suspension's turbidity was adjusted to that of 0.5 McFarland standards. A Muller-Hinton agar plate was prepared, and a sterile cotton swab was dipped into the suspension and wiped onto the plate's surface. Antibiotic discs (Himedia, Mumbai) were then placed on the agar plate with sterile forceps and gently pressed to ensure complete contact. After 24 h of incubation at 37 °C under aerobic and anaerobic conditions, the plates were read. Isolates were scored as susceptible, intermediate, or resistant to each antibiotic tested, according to CLSI [19] guideline, by measuring the zone of inhibition around the antibiotic disc, with intermediate results considered resistant [20]. Isolates showing resistance to three or more antibiotics were categorized as multiple drug resistance (MDR) phenotypes [21].

### Statistical analysis

The data was entered into a spreadsheet in Microsoft Excel and coded for statistical analysis. All statistical analyses were conducted using Stata 14.2 statistical software (Stata Corp LLC, 4905 Lakeway Drive, College Station, Texas). The cumulative incidence of uterine infection was calculated as the number of new events or cases of uterine infection during the study period divided by the total number of postpartum cows in the population at risk at the beginning of the study. Descriptive statistics were used to express the frequency and proportion of uterine infections and bacteria isolated from infected cows, as well as the proportion of isolated bacteria that developed resistance to tested antimicrobials. The chi-square (χ^2^) test was used to determine the association of uterine infection with the considered risk factors such as RFM, dystocia, abortion, milk fever or vaginal prolapse. Associations were deemed statistically significant if the calculated p-value was below 0.05.

## Results

### Cumulative incidence of uterine infection

Out of 236 cows monitored during the postpartum phase, 45 (19.1%) were found to have contracted uterine infection, which could be either metritis or clinical endometritis. The cumulative incidence of metritis was 11.4% (*n* = 27), while the cumulative incidence of endometritis was 7.6% (*n* = 18). It was observed that cows with uterine infection had a history of various reproductive health disorders. Retained fetal membrane (*p* < 0.001), dystocia (*p* < 0.001), and abortion (*p* = 0.004) were statistically significantly associated with uterine infection, while milk fever or hypocalcaemia (*p* = 0.051) and vaginal prolapse (*p* = 0.263) were not associated (Table 1).

**Table 1:** Association of selected risk factors with uterine infection

Risk factors		Cows examine	Uterine infection		χ^2^	*p*-value
			Frequency	Proportion		
RFM	Absent	185	8	4.32	120.58	0.000
	Present	51	37	72.54		
Dystocia	Absent	204	28	13.7	27.82	0.000
	Present	32	17	53.12		
Abortion	Absent	215	36	16.74	8.45	0.004
	Present	21	9	42.86		
Milk fever	Absent	230	42	18.26	3.82	0.051
	Present	6	3	50		
VP	Absent	234	44	18.8	1.25	0.263
	Present	2	1	50		

*RFM* Retained fetal membrane, *VP* Vaginal prolapse.

### Bacteria isolated

Out of 40 swab samples collected from cows with uterine infection (24 with metritis and 16 with endometritis) and subjected to bacterial isolation, 29 (72.5%) samples exhibited the presence of one or more bacteria, as determined by colony morphology and biochemical properties. The remaining 11 (27.5%) samples did not indicate any bacterial growth. Antibiotic treatment had already been administered to five cows out of the 45 with uterine infection, hence they were not included in the bacteriology sampling.

A total of 67 bacterial isolates were identified from the positive swab samples, belonging to 10 different genera. The bacteria identified included *Escherichia coli* (45%), coagulase positive staphylococci (CPS) (30%), *Klebsiella* spp. (22.5%), *Arcanobacterium pyogenes* (12.5%), *Fusobacterium* spp. (12.5%), *Enterobacter aerogenes* (12.5%), coagulase negative staphylococci (CNS) (12.5%), *Streptococcus* spp. (7.5%), *Salmonella* spp. (5%), *Proteus* spp. (5%), and *Pasteurella* spp. (2.5%), listed in descending order of frequency (Table 2). Among the swab samples, 25 contained mixed bacteria, with 12 samples having two different bacteria and 13 samples having three different bacteria.

**Table 2:** Bacterial species isolated from cows with uterine infection (*N* = 40)

Bacterial isolates	Frequency	Proportion (%)
*Escherichia coli*	18	45
*Coagulase positive staphylococci*	12	30
*Klebsiella* spp	9	22.5
*Arcanobacterium pyogenes*	5	12.5
*Fusobacterium* spp	5	12.5
*Enterobacter aerogenes*	5	12.5
*Coagulase negative staphylococci*	5	12.5
*Streptococcus* spp	3	7.5
*Salmonella* spp	2	5
*Proteus* spp	2	5
*Pasteurella* spp	1	2.5
Total 100%	67	

There were more instances of bacterial isolates in samples from cows with metritis as compared to cows with endometritis. Out of all the bacterial isolates, 43 (64.2%) were found in samples taken from metritis cases, and 24 (35.8%) were from endometritis cases. The types and frequency of bacteria varied in both cases. *E. coli* was the most commonly isolated bacteria in metritis cases, followed by *CPS* and *Klebsiella* spp. In endometritis cases, *CPS* was the leading bacteria, followed by *E. coli* and *Enterobacter aerogenes*. Additionally, *Pasteurella* spp was only isolated in endometritis cases (Table 3).

**Table 3:** Types and frequency of bacterial isolates from cases of metritis and endometritis

Bacterial Isolate	Metritis (*n* = 24)	Endometritis (*n* = 16)
*Escherichia coli*	14 (32.6)	4(16.7)
Coagulase positive staph	7(16.3)	5(20.8)
*Klebsiella* spp	6(14)	3(12.5)
*Arcanobacterium* pyogenes	4 (9.3)	1(4.2)
*Fusobacterium* spp	4(9.3)	1(4.2)
*Enterobacter aerogenes*	1(2.3	4(16.7)
Coagulase negative staph	2(4.65)	3(12.5)
*Streptococcus* spp	2(4.65)	1(4.2)
*Salmonella* spp	2(4.65)	1(4.2)
*Proteus* spp	1(2.3)	1(4.2)
*Pasteurella* spp	0(0.0)	1(4.2)
**Total bacterial isolates**	43	24

Values in the parenthesis are percentages.

### Antimicrobial susceptibility profiles of the bacterial isolates

Table 4 shows the results of the antimicrobial susceptibility test. It was found that the majority of bacteria, with the exception of *E. aerogenes*, *Streptococcus* spp., and *Salmonella* spp., had developed resistance to multiple antimicrobials. Multidrug resistance (MDR) was observed in *E. coli*, *Klebsiella* spp., *A. pyogenes*, and *Fusobacterium* spp., as they were resistant to three or more antimicrobials. Conversely, *CPS*, *CNS*, *Proteus* spp., and *Pasturella* spp. exhibited resistance to two antimicrobials. Among the six antimicrobials tested, gentamicin was found to be the most effective against all bacteria. All bacteria, except *CNS*, were fully susceptible to gentamicin. Among the *CNS* isolates, 50% were susceptible, while the other 50% showed intermediate resistance. Amoxicillin was the second most effective antimicrobial, with the majority of bacteria being susceptible to it. The exception was that 25% of *CPS* had developed resistance to it. Tetracycline was the least effective antimicrobial, as seven out of the eleven bacteria displayed varying degrees of resistance to it. Ampicillin was the second least effective antimicrobial, with five of the tested bacteria developing different levels of resistance.

**Table 4:** Antimicrobial susceptibility patterns of bacteria isolated from uterine discharge from cows with postpartum problems

**Type and number of isolates tested**	Antimicrobial tested																	
	**T10μg/disc**			**Ch30μg/disc**			**G10μg/disc**			**St10μg/disc**			**Am20μg/disc**			**AMP25μg/disc**		
	**R**	**I**	**S**	**R**	**I**	**S**	**R**	**I**	**S**	**R**	**I**	**S**	**R**	**I**	**S**	**R**	**I**	**S**
*E.coli* (6)	50	0	50	0	16.7	83.3	0	0	100	33.3	16.7	50	0	0	100	50	0	50
*CPS* (4)	0	0	100	0	0	100	0	0	100	0	50	50	25	0	75	75	25	0
*Klebsiella* (3)	100	0	0	66.7	0	33.3	0	0	100	33.3	33.3	33.3	0	0	100	0	0	100
*A. pyogenes* (2)	50	0	50	50	50	0	0	0	100	50	50	0	0	50	50	50	50	0
*Fusobacterium* (3)	33.3	0	66.7	66.7	0	33.3	0	0	100	0	0	100	0	0	100	33.3	0	66.7
*E.aerogenes* (2)	0	0	100	0	0	100	0	0	100	0	50	50	0	0	100	0	0	100
*CNS* (2)	50	0	50	0	0	100	0	50	50	50	50	0	0	100	0	0	0	100
*Streptococus* (1)	0	100	0	0	0	100	0	0	100	0	0	100	0	0	100	0	0	100
*Salmonella* (1)	0	0	100	0	100	0	0	0	100	0	0	100	0	0	100	0	0	100
*Proteus* (1)	100	0	0	0	100	0	0	0	100	100	0	0	0	100	0	0	0	100
*Pasteurella* (1)	100	0	0	0	0	100	0	0	100	0	0	100	0	0	100	100	0	0
**Total (26)**	**42.3**	**3.8**	**53.8**	**19.2**	**15.4**	**65.4**	**0**	**3.8**	**96.2**	**23.1**	**26.9**	**50**	**3.8**	**15.4**	**80.8**	**34.6**	**7.7**	**57.7**

*R Resistant, *I*  Intermediate,* S *Susceptible, *T*  Tetracycline, *Ch* Chloramphenicol, *G* Gentamicin*, St *Streptomycin,* Am* Amoxicillin,* Amp* Ampicillin,* CPS* Coagulase positive staphylococci*, CNS* Coagulase negative staphylococci

Among the *E. coli* isolates, a significant level of resistance was noted against commonly used antimicrobial agents, specifically tetracycline (50%) and ampicillin (50%). In contrast, a relatively lower resistance rate was observed against streptomycin (33.3%).Nonetheless, the *E. coli* isolates were found to be highly susceptible to gentamicin (100%), amoxicillin (100%), and chloramphenicol (83.3%).

Coagulase positive staphylococci exhibited a high level of resistance to ampicillin (75%) and a lower rate of resistance rate to amoxicillin (25%). However, it displayed high sensitivity to tetracycline (100%), chloramphenicol (100%), gentamicin (100%), ampicillin (75%)and streptomycin (50%).

*Klebsiella* spp were highly resistant to tetracycline(100%) and chloramphenicol (66.7%) and a lower rate of resistance to streptomycin (33.3%), but all isolates were susceptible to gentamicin, amoxicillin and ampicillin. Of the total isolates of *A. pyogenes*, 50%were resistant to tetracycline, chloramphenicol, streptomycin and ampicillin, but 100% of them were susceptible to gentamicin.

Coagulase negative staphylococci exhibited resistance to tetracycline (50%), gentamicin (50%) and streptomycin (50%), while all isolates were susceptible to chloramphenicol and ampicillin. *Fusobacterium* spp developed varying degree of resistance to tetracycline (33.3%), ampicillin (33.3%), and chloramphenicol (66.7%) but it was 100% susceptible to gentamicin, streptomycin, and amoxicillin (Table 4).

## Discussion

The study found that a significant proportion of postpartum cows (19.1%) in the population were affected by uterine infections. Our study specifically examined the risk of developing metritis and endometritis in dairy cows. Metritis had a cumulative incidence of 11.4%, while endometritis had a cumulative incidence of 7.6%. These results suggest that both metritis and endometritis are significant concerns among dairy cows, although metritis appears to be slightly more common. Since the postpartum period is critical to a cow's future lactation and fertility, the observation of a high incidence of uterine infections in the cows studied has significant implications for farmers, veterinarians, and policy makers. Timely diagnosis and treatment by veterinarians can protect cows' welfare, while policymakers can create a supportive environment for preventive measures. Good management practices are crucial, including proper hygiene, avoiding trauma to the genital tract, reducing stress, feeding animals appropriately, and monitoring cow health. Educating dairy farmers about proper herd health is also important [22].

The cumulative incidence of metritis observed in the current study is consistent with previous studies conducted in Ethiopia, which reported cumulative incidence ranging from 5.6% to 16.63% [13, 14]. However, other countries have reported higher cumulative incidence, ranging from 20 to 40% [23–27]. The cumulative incidence of endometritis in the present study is lower than previous reports from other countries, such as 27% in the UK [28] and 67.2% in Rwanda [29]. These differences in cumulative incidence may be attributed to various factors, including herd management, healthcare practices, breed compositions, environmental conditions, or diagnostic methods.

The present study revealed a significant association between retained placentas (RP), dystocia, and abortion with the cumulative incidence of uterine infection. In line with the current finding, several previous reports [16, 28, 30, 31] indicated that uterine infection, such as metritis and endometritis, can be caused by various factors, including primiparity, season, dystocia, twins, RP, stillbirths, abortions, and uterus prolapse. These reproductive disorders can lead to a delay in the complete clearance of lochia and the maintenance of cervical patency, creating favourable conditions for bacterial invasion and growth [28, 32, 33]. Additionally, unsanitary housing environments can increase the risk of infection, even when the uterine defense mechanisms are intact [17]. Overall, these findings indicate that reproductive disorders, combined with unhygienic assistance during dystocia, serve as risk factors for uterine infection.

The present study successfully identified 62 bacterial isolates from 10 genera, both aerobic and anaerobic. Among these, *E. coli* was the most prevalent bacteria, present in 45% of uterine swabs. It was the primary cause of metritis and the second most common cause of endometritis. These findings are consistent with other studies that have also listed *E. coli* as a leading cause of post-calving uterine infections in dairy cows [4, 23, 32, 34, 35]. According to the literature, *E. coli* is a primary pathogen that can invade the uterus during calving or contaminated procedures. It is typically more common in the early postpartum period and appears to pave the way for later infection with other bacterial species or viruses [9]. In addition to *E. coli*, other frequently isolated bacteria included *CPS*, *Klebsiella* spp., *A. pyogenes*, *Fusobacterium* spp., *E. areogenes* and *CNS*. These findings are consistent with numerous previous studies worldwide, which have also reported the isolation of these bacteria frequently from the uterus of postpartum cows and their involvement in the pathogenesis of uterine disease [10, 17, 36–40]. However, different studies do not uniformly identify a single or group of bacteria as the main cause of uterine infection in cows. Rather, they acknowledge mixed contamination as the cause. The lack of agreement in the literature may be partly attributed to the timing of the isolations, as well as the methods used to collect, transport, and process the uterine samples for bacterial isolation [40]. It has been reported that more than 90% of cows carry microorganisms in their uterus during the postpartum period, regardless of any signs of illness [17, 41]. However, in the present study, the isolation of the reported bacteria from cows with uterine infection may be due to heavy bacterial colonization following dystocia, retained fetal membranes, poor hygiene, and weak uterine defense mechanisms [42, 43].

More than a quarter (27.5%) of samples taken from cows with typical clinical signs of metritis and endometritis were bacteriologically negative. This could be attributed to various factors such as the low sensitivity of the culture-based testing method used, the use of undisclosed antibiotics, errors in sample collection and transportation, or the spontaneous recovery of the cases and the presence of some fastidious bacteria [40]. As a result, further research using cutting-edge culture-independent molecular methods such as MALDI-TOF mass spectrometry, is necessary to study uterine microbiota/pathogens.

Our investigation into the antimicrobial susceptibility profile of bacteria isolated from the uterus of postpartum dairy cows revealed that eight out of the eleven bacterial species exhibited varying degrees of resistance to two or more tested antimicrobial drugs. All antimicrobials tested, except gentamycin showed AMR. Furthermore, the present study highlighted the urgent concern of MDR, which has been observed in *E. coli*, *Klebsiella* spp., *A. pyogenes* and *Fusobacterium* spp. These bacteria have demonstrated resistance to three or more antimicrobials, albeit with different patterns of MDR. The most severe case of MDR was observed in *A. pyogenes,* which developed resistance to four antimicrobials: tetracycline, chloramphenicol, streptomycin and ampicillin. *E. coli*, *Klebsiella* spp. and *Fusobacterium* spp exhibited resistance to three antimicrobials each: tetracycline––streptomycin–ampicillin, tetracycline–chloramphenicol–streptomycin, and tetracycline–chloramphenicol–ampicillin, respectively. These findings highlight the limited treatment options available for these bacterial species. Similar MDR profiles have been reported in other studies where the same bacteria were isolated from the uterus of dairy cows [12, 15, 32, 35]. However, in contrast to our findings, Santos et al. [44] reported excellent activity of tetracycline and streptomycin against *E. coli* isolates.

The study found that among the tested antimicrobial agents, gentamicin was the most effective, with 96.2% of all bacterial isolates being susceptible. Amoxicillin was the second most effective, 80.8% of bacterial isolates being susceptible. Therefore, dairy farmers should consider using these antimicrobials to treat acute or chronic uterine infections. Previous studies have also shown gentamicin to be highly effective against Gram negative bacteria [32, 45, 46]. However, in contrast to our results, some studies found resistant to gentamicin and amoxicillin in cows with metritis and endometritis [15, 47]. It is important to note that while gentamicin and amoxicillin have shown promising efficacy against a wide range of bacterial isolates, the decision to use these antimicrobials should be made in consultation with a veterinarian as the effectiveness of antimicrobial agents can vary depending on various factors such as geographical location, bacterial resistance patterns, and individual animal health conditions [48]. In this study, tetracycline was the least effective antimicrobial agent, with seven out of the eleven bacterial species showing resistance. Tetracyclines are widely applied in veterinary practice, including for treating uterine infections like metritis [49]. In general, and specifically in the present study areas of Ethiopia, long-acting oxytetracycline in systemic form is widely employed by veterinary practitioners to treat uterine infections due to the absence of intrauterine antibiotics in the market. In some cases, dairy farmers themselves administer the antibiotics. Therefore, it is believed that the indiscriminate use of tetracycline, which is easily accessible in local markets by non-professionals may have contributed to the emergence of tetracycline-resistant bacterial species observed in this study.

## Conclusions

The study found that a significant number of cows in the population were affected by uterine infections, namely metritis and endometritis. The research identified various species of bacteria, both aerobic and anaerobic, that cause uterine infections, with *E. coli*, coagulase-positive staphylococci, and *Klebsiella* spp being the most frequently isolated. The bacteria isolated from infected cows demonstrated varying degrees of resistance to many of the antimicrobials tested, with MDR occurring in *E. coli*, *Klebsiella* spp., *A. pyogenes* and *Fusobacterium* spp. The worst AMR profile was found for tetracycline, with seven of the eleven bacteria showing varying levels of resistance to it. While most of the antimicrobials developed resistance, gentamicin proved to be effective for nearly all bacterial isolates. The study emphasizes the importance of early diagnosis and treatment of uterine infections to prevent adverse effects on the health and productivity of cows. Additionally, responsible use of antimicrobials is necessary to prevent the emergence of antimicrobial resistance, and further research is needed to understand the mechanisms of antimicrobial resistance. It is also important to raise awareness among dairy farmers about good herd health management practices and the negative consequences of indiscriminate antimicrobial use.

## Data Availability

All data generated or analyzed during this study are included in this article and are available from the corresponding author upon reasonable request.
